# Revisiting Drug Development Against the Neglected Tropical Disease, Amebiasis

**DOI:** 10.3389/fcimb.2020.628257

**Published:** 2021-02-24

**Authors:** Manish T. Shrivastav, Zainab Malik, Somlata

**Affiliations:** Multidisciplinary Centre for Advanced Research and Studies, Jamia Millia Islamia, New Delhi, India

**Keywords:** protozoan parasite, amebiasis, drug targets, metabolic kinases, protein kinases, proteases, metabolic enzymes, drug repurposing and discovery

## Abstract

Amebiasis is a neglected tropical disease which is caused by the protozoan parasite *Entamoeba histolytica*. This disease is one of the leading causes of diarrhea globally, affecting largely impoverished residents in developing countries. Amebiasis also remains one of the top causes of gastrointestinal diseases in returning international travellers. Despite having many side effects, metronidazole remains the drug of choice as an amebicidal tissue-active agent. However, emergence of metronidazole resistance in pathogens having similar anaerobic metabolism and also in laboratory strains of *E. histolytica* has necessitated the identification and development of new drug targets and therapeutic strategies against the parasite. Recent research in the field of amebiasis has led to a better understanding of the parasite’s metabolic and cellular pathways and hence has been useful in identifying new drug targets. On the other hand, new molecules effective against amebiasis have been mined by modifying available compounds, thereby increasing their potency and efficacy and also by repurposing existing approved drugs. This review aims at compiling and examining up to date information on promising drug targets and drug molecules for the treatment of amebiasis.

## Introduction

Amebiasis is a disease caused by the protozoan parasite *Entamoeba histolytica* and is a major public health crisis in developing countries (Jeelani and Nozaki, 2014). The global burden lies in tropical and subtropical countries suffering from poor sanitation facilities (Shirley et al., 2018). As per WHO, *Entamoeba histolytica* infects approximately 50 million people worldwide and causes the death of around 100,000 people annually (Ben Ayed, 2015). The parasite is among the top 15 causes of diarrhoea in children under the age of two years and thus hampers their mental and physical growth (Haque et al., 2006). Around 90% of the infected individuals are asymptomatic. The factors that turn the parasite virulent in remaining individuals are still unknown but gut microbiota has been associated as one of the factor for triggering virulence (Padilla-Vaca et al., 1999). The symptoms of amebiasis range from mild diarrhoea, dysentery to invasive colitis, liver abscesses and rare lung and/or brain abscesses (Zulfiqar et al., 2020). Children below the age of five years are more prone to infection (Haque et al., 2006; Gebretsadik et al., 2018). Humans are the only known hosts for this parasite (Brock and Bricker, 2018). This microaerophilic parasite follows a two-stage life cycle consisting of a non-invasive but infective cyst form which is dormant but highly resistant to harsh external environment conditions (Saidin et al., 2019) and an invasive but non-infective trophozoite which is active inside the host but cannot survive in the external environment (Guevara et al., 2019). Trophozoites reside in the intestine where they endocytose the mucosal cells and commensal bacteria. Stool microscopy, ELISA and PCR are the methods of diagnosis for amebiasis with PCR being the most preferred one (Shirley et al., 2018; Guevara et al., 2019; Saidin et al., 2019).

The most commonly used medication against amebiasis is metronidazole (Mtz). Mtz is activated by thioredoxin reductase and possibly by ferredoxin to produce a nitroradical anion and a nitroimidazole compound on subsequent reduction. These two metabolites exert toxic effects on the trophozoites. The cytotoxic effects that follow, include breakage and destabilization of DNA helix leading to inhibition of protein synthesis which proves to be fatal for the parasite (Weir and Le, 2020). on the other hand, *E. histolytica* has also exhibited resistance to metronidazole under laboratory conditions (Wassmann et al., 1999) and a higher tolerance to Mtz by clinical isolates of *E. histolytica* has been reported from India (Iyer et al., 2014). Also, protozoan parasites having similar metabolism like *Giardia* have exhibited resistance against Mtz in clinical isolates (Iyer et al., 2014). Besides the increasing threat of resistance, Mtz therapy has side effects ranging from minor ones like nausea, vomiting, headaches, metallic or bitter taste in the mouth to serious ones like ataxia, anorexia and skin rashes (Weir and Le, 2020). Hence, there is a need to look out for new drug targets and alternative strategies for the treatment of amebiasis.

**Figure 1:**
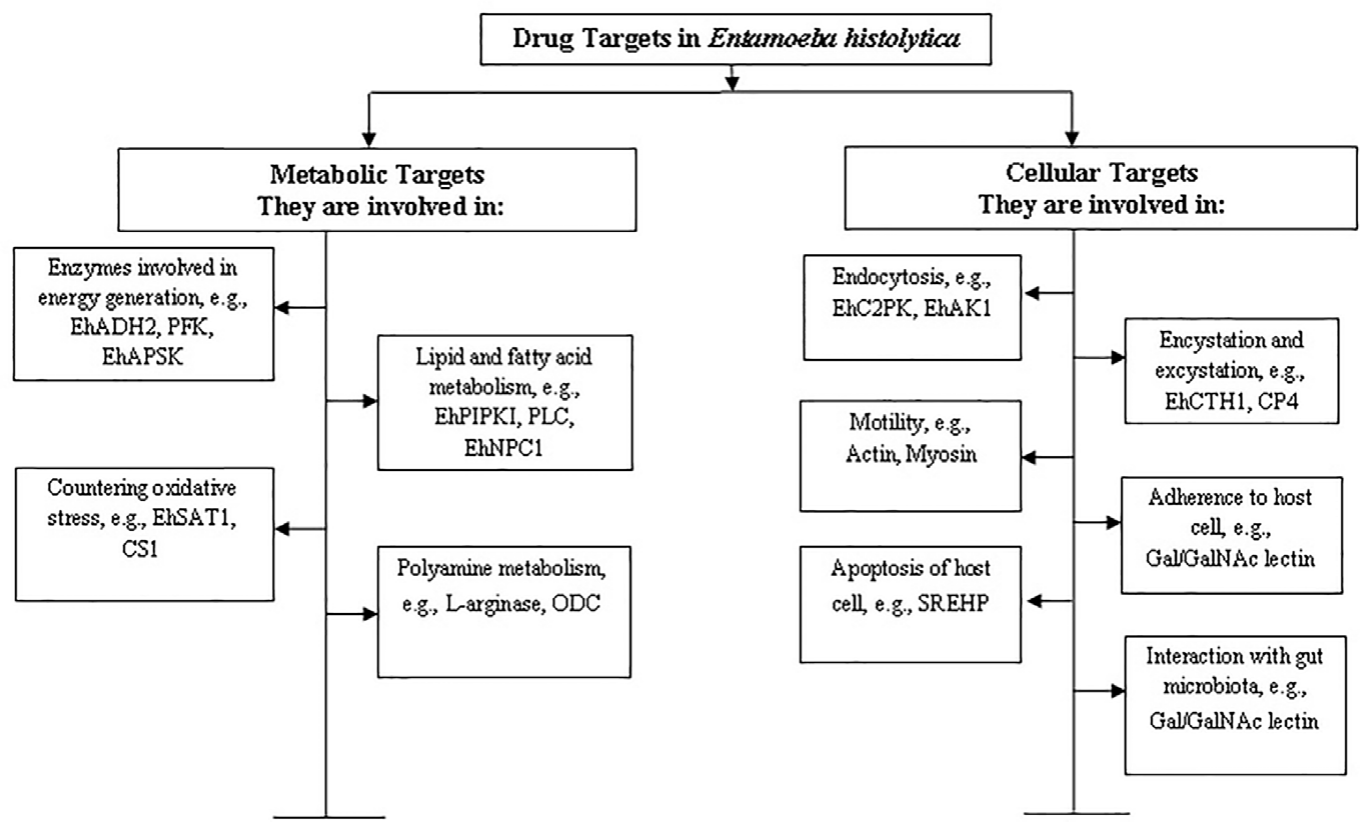
Categorization of drug targets in *Entamoeba histolytica*. The targets have been categorised on the basis of their function in metabolic or cellular processes. Some examples of proteins involved in the corresponding processes have been mentioned and further discussed in the review.

*E. histolytica* is a microaerophilic and a highly motile organism which has been found to survive in the host system through unique metabolic and cellular processes. This review focuses on compiling information on promising drug targets found so far in *E. histolytica* along with analysing their potential in the light of recent developments. On the basis of available literature, we have categorised the drug targets into two broad categories: (a) metabolic and (b) cellular. An outline of these targets has been described in **Figure 1**.

Being a microaerophilic parasite, the *E. histolytica* metabolism is significantly different from humans and hence offers a promising avenue for identifying targets for anti-amebic drugs. Examples of metabolic drug targets include the biomolecules and/or enzymes involved in L-cysteine synthesis (a major anti-oxidant for the parasite), in sulphur synthesis and transport to and from mitosomes (a primitive form of mitochondria), in energy generation pathways like glycolysis such as phosphofructokinase and NADH kinase etc.

Cellular drug targets, on the other hand are those which are involved in essential processes like endocytosis, motility, cell division etc. Pathogenesis by the trophozoites largely depends upon two endocytic processes—phagocytosis and trogocytosis (Ralston, 2015a). Any defect leading to a decrease in these endocytic capabilities leads to the loss of pathogenesis and virulence potential of the parasite (Christy and Petri, 2011). The research so far, has revealed many biomolecules which are involved in pathways unique to this organism and not present in the host system. For example, CaBPs (calcium binding proteins), C2PK (C2 domain containing protein), EhAK1, APSK (an unconventional alpha kinase), etc. (Mansuri et al., 2014; Babuta et al., 2020). Further, probiotics, drugs and small molecules which act on the gut microbiota and alter the parasite biology can prevent invasion and subsequent pathogenesis, thereby offering an interesting alternative for the treatment of amebiasis and other parasitic intestinal infections (Burgess and Petri, 2016).

## Metabolic Drug Targets

*Entamoeba histolytica* is a primitive eukaryote which has cellular organelles and metabolic pathways that greatly differ from those in higher eukaryotes such as humans (Bruchhaus and Tannich, 1994). For example, it possesses mitosomes which are primitive counterparts of mitochondria and carry out substrate-level phosphorylation for energy generation (Hjort et al., 2010). Also, being microaerophilic, the parasite requires very low levels of oxygen and lacks aerobic metabolic pathways like tricarboxylic acid (TCA) cycle and oxidative phosphorylation (Anderson and Loftus, 2005). However, the parasite is capable of moulding its metabolic pathways as per its external milieu and requirements (Anderson and Loftus, 2005). Thus, the unique metabolic pathways operating in the parasite open a new window of opportunities for identifying unique and effective drug targets.

### Drug Targets Involved in Energy Generation Pathways

#### Alcohol Dehydrogenase

Alcohol dehydrogenase 2 (EhADH2) is a 95-kDa bifunctional NAD^+^-linked and Fe^2+^-dependent enzyme present in *E. histolytica*. It is a very crucial enzyme as it exhibits activities of both, alcohol dehydrogenase (ADH) and aldehyde dehydrogenase (ALDH) (Espinosa et al., 2016). The parasite lacks Krebs cycle and relies on fermentation of glucose to ethanol for energy generation (Pineda et al., 2015). The two final steps of the glycolytic pathway are catalysed by this enzyme - the conversion of acetyl-CoA to acetaldehyde and the reduction of acetaldehyde to ethanol. The EhADH2 protein has been reported to be a fusion protein, with N-terminal ALDH and C-terminal ADH domains. EhADH2 is required for the survival and growth of trophozoites (Espinosa et al., 2001) and thus is a potential drug target. EhADH2 is homologous to the trifunctional AdhE protein of *E. coli* (Yang et al., 1994). The enzyme has also been reported to complement the function of AdhE in a mutant strain of *E. coli*, facilitating the screening of anti-amoebic compounds (Yong et al., 1996). Cyclopropyl carbinols (CPC) and cyclobutyl carbinols (CBC) hinder the growth and viability of trophozoites by affecting the ALDH and ADH activities of EhADH2 (Espinosa, 2004). These findings point to the usefulness of EhADH2 as a promising drug target.

#### Phosphofructokinase (PFK) and Related Enzymes

*E. histolytica* trophozoites largely depend on glycolysis for meeting their energy needs as the enzymes involved in Krebs cycle are absent. One of the important enzymes, phosphosphofructokinase (PFK) is coded by the genome of this parasite. The amoebic PFK is different from its human counterpart as it uses pyrophosphate as its cofactor unlike the human PFK which is ATP-dependent (Pineda et al., 2015). Targeting the sole energy generating pathway of this parasite seems to be a very efficient strategy to kill the parasite in the host system, especially when the enzyme involved has a different mode of operation for the same biochemical reaction. This ensures specificity of the drug for the parasite while avoiding its side effects in the host system. The enzyme is required for phosphorylation of fructose-6-phosphate during glucose metabolism and hence plays a significant role in the energy generation pathway (Mertens, 1993). Phosphofructokinase in this parasite has been shown to be competitively inhibited by six different bisphosphonate inhibitors (Eubank and Reeves, 1982) as well as pyrophosphate analogues (Bruchhaus et al., 1996) and thus is a well-established drug target.

Other enzymes in this parasite which also utilise pyrophosphate as a co-factor are PEP carboxytransphosphorylase, pyruvate phosphate dikinase, and pyrophosphate-acetate kinase. These have also been shown to be inhibited by bisphosphonates (Mertens, 1993; Varela-Gómez et al., 2004; Chiba et al., 2015) but the efficacy of these targets remains to be explored and tested experimentally.

Recently, another enzyme Triosephosphate isomerase (EhTIM) has been targeted by the compound 5,5′-[(4-nitrophenyl)methylene]bis(6-hydroxy-2-mercapto-3-methyl-4(3H)-pyrimidinone). EhTIM is involved in the interconversion of glyceraldehyde 3-phosphate to dihydroxyacetone. The compound has been reported to have IC50 of 18.4 µM in *in vitro* studies which is a good indicator for further assessment of this compound as an anti-amoebic drug and of EhTIM as a potential drug target (Vique-Sánchez et al., 2020).

#### APS Kinase

Adenosine 5′-phosphate kinase (EhAPSK) is a key enzyme in sulphur metabolism. *E. histolytica*, being microaerophilic, has a highly diverged form of mitochondria, known as mitosomes which are predominantly involved in sulphate activation (Makiuchi and Nozaki, 2014). In an immunofluorescence assay, it has been observed that three proteins were involved in sulphate activation- ATP sulphurylase, APS kinase, and inorganic pyrophosphatase. Also, a sodium/sulphate symporter present in the mitosome membrane was found to be responsible for sulphate uptake (Mi-ichi et al., 2009). Sulphur metabolism is an excellent target because it is involved in many pleiotropic roles related to the maintenance of parasite’s life-cycle through sulpholipids (Mi-ichi et al., 2017). Cholesteryl sulphate (CS), the end product of sulphur activation pathway is known to play an important role in encystation and is very crucial for differentiation (Mi-ichi et al., 2015). Mi-ichi et al. (2019) have identified three APSK inhibitors on the basis of *in vitro* and *in silico* analysis by screening a library of 400 compounds. The compounds identified were 2-(3-flurophenoxy)-N-[4-(2-pyridyl)thiazol-2-yl] acetamide, 3-phenyl-N-[4-(2-pyridyl)thiazol-2-yl]-imidazole-4-carboxamide and auranofin (Mi-ichi et al., 2019). The APSK inhibitors were reported to halt the proliferation of *E. histolytica* trophozopites as well as cyst formation (observed in *E. invadens* as a model system). All these compounds decreased the synthesis of sulpholipids in a dose dependent manner (Mi-ichi et al., 2019). The already established drug auranofin which is known to target thioredoxin (Trx) and thioredoxin reductase (TrxR) was also found to inhibit APSK (Mi-ichi et al., 2019). These compounds inhibited proliferation as well as cyst formation, processes which are directly associated with the pathogenesis and transmission of the parasite. Hence, APSK and its inhibitors are justified candidates for alternate anti-amebiasis therapy.

### Lipid Metabolism

Pathogenesis by the parasite occurs in a sequential manner - mucus degradation, adherence to host epithelial cells, cell lysis through toxins and lytic agents followed by phagocytosis of dead or apoptotic cells (Espinosa-Cantellano and Martinez-Palomo, 2000). It should be noted that all the aforementioned processes are dependent on lipids present in membranes (Espinosa-Cantellano and Martinez-Palomo, 2000; Byekova et al., 2010; Das and Nozaki, 2018). In addition, after the phagocytosis of host cells, their processing takes place through complex vesicular trafficking network  (Das and Nozaki, 2018), as the parasite lacks most of the organelles found in higher eukaryotes  (Smith and Guillen, 2010). This extensive vesicle trafficking network again depends on various types of lipids and their interconversion. The parasite’s lipid composition differs from that of host cells  (Aley et al., 1980; Cerbón and Flores, 1981; van Meer and de Kroon, 2011), the major difference being higher ceramide and phosphatidylcholine (PC) content and lower phosphoinositide (PI) content. Also cholesterol:phospholipid ratio of amebic cells is 0.8 as compared to mammalian cells, which is 0.3 in whole cells  (Aley et al., 1980; Vance, 2015). Considering the peculiar lipid composition of the parasite and a repertoire of 27 putative unexplored enzymes involved in lipid metabolic pathway (Castellanos-Castro et al., 2020), the parasite offers a promising avenue for anti-amebic drug targets.

#### Metabolism of Exogenous Lipids

*E. histolytica* does not synthesise all the lipids required by it but is dependent on the absorption of many from the extracellular milieu  (Sawyer et al., 1967; Singh et al., 1971; Serrano-Luna et al., 2010; Bolaños et al., 2016). Lipases process such lipids along with about 22 homologues of lipid transfer proteins. Out of these proteins, 15 contain START-domain (required for binding to sterols, phospholipids and ceramides), 4 contain ORD-domain (required for binding to phosphatidylinositol-4 phosphate, sterols), 2 contain Sec14-domain (required for binding to phosphatidylcholine (PC) and phosphatidylinositides) and 1 protein contains PRELI-domain [required for transferring phosphatidic acid (PA)]  (Das et al., 2002; Das and Nozaki, 2018). However, the lipid transport machinery of *E. histolytica* has just begun to be explored and this might lead to discovery of drug targets specific to this parasite. Blocking the lipid transport is expected to affect plasma membrane stability, endocytic processes and cyst formation.

#### Metabolism of Phosphatidylcholine (PC), Phosphatidylethanolamine (PE) and Sphingolipids (SL)

Phosphatidylcholine (PC) and phosphatidylethanolamine (PE) are the most abundant glycerophospholipids in *E. histolytica*  (Aley et al., 1980; Cerbón and Flores, 1981). The lipids are synthesized through Kennedy pathway, which has been experimentally verified to be present in the parasite. The Kennedy pathway is upregulated during L-cysteine deprivation in (Husain et al., 2010; Jeelani and Nozaki, 2014) which leads to an increased concentration of lipids along with that of an unconventional lipid phosphatidyl isopropanolamine  (Husain et al., 2010). Further, SL metabolism is also known to be linked with PC and PE metabolism  (Redirection of sphingolipid metabolism toward de novo synthesis of ethanolamine in Leishmania, 2017; Zufferey and Mamoun, 2002; Peacock et al., 2007). However, sphingomyelin (SM) is present at very low levels in the trophozoites under normal conditions  (Aley et al., 1980; Cerbón and Flores, 1981) but it cannot be ruled out that the parasite fulfills its PE requirement through SL metabolism and Kennedy pathway (Gulati et al., 2015) as its genome codes for a putative sphingomyelin phosphodiesterase (EHI_007460) and another similar enzyme (EHI_040600)  (Njoya et al., 2014; Castellanos-Castro et al., 2020). These enzymes carry out the conversion of SM to phosphocholine which is then directed to the Kennedy pathway. The deactylated form of PC, lysophosphatidylcholine, is a lytic agent released by the amoeba upon encountering host cells. Also, like bacterial lipopolysaccharides (LPS), *E. histolytica* possesses lipophosphopeptideglycan (LPPG) which is related to its virulence with PE being the ethanolamine group donor (Raetz et al., 2007; Wong-Baeza et al., 2010). Furthermore, as the ceramide proportion in trophozoites is higher, it is assumed to provide stability and plasticity to the parasite. The CEP in the plasma membrane is resistant to hydrolysis and protects the trophopzoites from proteases secreted by the host tissue during invasion  (Cerbón and Flores, 1981). Ceramide biosynthesis has not been studied in the parasite but Mfotei Njoya et al. (2014) have pointed to the possibility of treatment with extract of the plant, *Codiaeum variegatum* which causes defects in lipid trafficking and ceramide accumulation in trophozoites. This ceramide accumulation decreases adhesion to the host cells due to disruption of lipid rafts (Njoya et al., 2014). Enzymes involved in ceramide synthesis have also been shown to be inhibited in *Dictyostelium discoideum* by cisplastin (Min et al., 2005), and in *Plasmodium falciparum* and *Plasmodium knowlesi* by sphingolipid analogues (Meyer et al., 2012). In *Dictyostelium discoideum*, sphingosine kinase mutants showed altered growth rates (Min et al., 2005) while in *Plasmodium falciparum* and *Plasmodium knowlesi*, there was retardation in growth and development (Meyer et al., 2012). Hence, blocking the transfer, conversion, and biosynthesis of these lipid molecules might lead to reduced virulence and pathogenesis. This aspect of the anti-amebic drug targets has been overlooked and offers to be a promising avenue.

#### Metabolism of Phosphatidylinositol (PI) and Phosphoinositides

Phosphatidylinositol (PI) and phosphoinositides play an important role in adhesion, eliciting the host immune response, virulence and signalling. The inositol group forms a part of LPG (lipopeptidoglycan) and LPPG (lipophosphopeptideglycan) which also aid in adhesion and cytotoxicity (Moody et al., 1998; Mann, 2002; Stanley et al., 1992). The concentration of LPPG is higher in virulent strains as compared to avirulent ones  (Bhattacharya et al., 2000). The LPPG molecules are reported to be immunogenic and antibodies have been found in the mice models and humans infected by the parasite (Acosta-Altamirano et al., 1986; Maldonado-Bernal et al., 2005; Wong-Baeza et al., 2010). Although the protective nature of the antibodies has not been characterized in detail, these molecules offer to be a target for vaccine generation.

PI contains an inositol group which gets phosphorylated at positions 3, 4, and 5 separately or in combination giving rise to seven different compounds  (Balla, 2013). Phosphoinositides are known to play important role in endocytosis, motility and other cellular functions. PI3P (Phosphatidylinositol-3-phosphate), PI(4,5)P_2_ (Phosphatidylinositol-4,5-bisphosphate), and PI(3,4,5)P_3_ (Phosphatidylinositol-3,4,5-trisphosphate) aid the formation of phagocytic cup  (Powell et al., 2006; Byekova et al., 2010). EhPIPKI catalyzes the synthesis of PI(4,5)P_2_ which maintains cell shape by binding to the cytoskeleton  (Sharma et al., 2019). PI(4,5)P_2_ might also be involved in parasite motility  (Das and Nozaki, 2018). The enzyme has also been shown to be inhibited in *Plasmodium falciparum* resulting in blockage of hemoglobin transport to its food vacuole and its eventual inability to obtain amino acids crucial for survival (Vaid et al., 2010). Also, the enzyme phospholipase C (PLC) hydrolyzes PI(4,5)P_2_ and PI(3,4,5)P_3_ yielding Ca^2+^, IP_3_ and DAG which are important signalling molecules  (Kortholt et al., 2007). Upon coming in contact with fibronectin, the intracellular concentration of PLC in trophozoites increases suggesting its role in locomotion  (Meza, 2000).

Although PI phosphorylation and dephosphorylation have been recently started to be explored, it is tentative to assume that detailed analysis of kinases and phosphatases might lead to a drug target of choice. The kinases involved in PI machinery of *Entamoeba* are distinct from their mammalian counterparts, which is helpful in making such an assumption (Nakada-Tsukui et al., 2019).

#### Cholesterol Metabolism

Cholesterol is an important molecule necessary for maintaining organization of lipid membranes and associated structures like lipid rafts  (Schroeder, 1990). Lipid rafts are like platforms that converge the signalling events during host-pathogen interaction and invasion  (Bansal et al., 2005). Mammalian cells (enterocytes and hepatic cells) are capable of synthesizing cholesterol *de novo*  *(* Ikonen, 2008). However, *E. histolytica* derives its cholesterol from intestinal and hepatic cells when it is present in the host gut and from the extracellular milieu when it is cultured  (Bansal et al., 2005). The parasite genome so far has not shown the presence of any gene related to sterol synthesis. This further supports the notion that the parasite takes cholesterol from its external milieu and transports it *via* vesicular network  (Bolaños et al., 2016; Castellanos-Castro et al., 2020). Bolanos et al. (2016) have reported EhNPC1 and EhNPC2 proteins to be involved in cholesterol transport in the parasite. In their study, the downregulation of EhNPC1 and 2 led to lower intracellular cholesterol levels and a decrease in phagocytic capabilities (Bolaños et al., 2016). These enzymes have also been shown to be inhibited in *P. falciparum* resulting in hampering of its plasma membrane and malformed digestive vacuoles (Istvan et al., 2019). Since, cholesterol is important for membrane organization and dynamics which in turn are important for endocytosis and motility, EhNPC1 and 2 can be considered promising targets for developing anti-amebiasis therapy.

#### Metabolism of Acetyl-CoA and Fatty Acids

Pantothenate kinase is required for the synthesis of CoA and has been identified in *E. histolytica* (Nurkanto et al., 2018a). It catalyses the first step which is activation of carbonyl group and an acyl carrier during the formation of fatty acids. When the pantothenate kinase gene was silenced in trophozoites, their CoA concentrations decreased and their growth was slowed down (Nurkanto et al., 2018a). Also, inhibitors of pantothenate kinase in *Mycobacterium tuberculosis* (Reddy et al., 2014) and *Bacillus anthracis* (Shapiro et al., 2019) have already been well studied, which makes it feasible to assume the possibility of pantothenate kinase in *E. histolytica* being a drug target.

Acetyl-CoA is an important cofactor involved in approximately 9% of all the biochemical activities that have been identified so far. These include amino acid synthesis, glycogen metabolism, nucleic acid metabolism and fatty acid metabolism (Strauss, 2010; Nurkanto et al., 2018b). CoA synthesis in *E. histolytica* is carried out in a four step process (Nurkanto et al., 2018b). Pantothenate kinase (PanK) catalyses the first step while dephospho-CoA kinase (DPCK) catalyses the last step (Leonardi et al., 2005; Nurkanto et al., 2018a). Both enzymes are ATP dependent and are essential in many organisms including *E. histolytica* (Hart et al., 2017). The metronidazole target - pyruvate:ferredoxin oxidoreductase is also involved in acetyl-CoA production (Samarawickrema, 1997). Hence, given the importance of CoA metabolism, the enzymes involved hold a promise for being efficient drug targets. The *E. histolytica* genome codes for two copies of DPCK, namely EhDPCK1 and 2. EhDPCK1 shows 31% similarity to human orthologs, while that for EhDPCK2 is 34% (Nurkanto et al., 2018a). The silencing of DPCK1 and 2 leads to an overall decrease in intracellular CoA concentrations and a decrease in growth rate. The silencing of DPCK2 causes more defects in cell growth and metabolite profile as compared to that of DPCK1. Also, the silencing of DPCK1 leads to a decrease in citrate, ornithine and S-adenosyl L-methionine along with that of CoA. On the other hand, silencing of DPCK2 leads to a decrease in comparably larger number of metabolites such as pantothenate, acetyl-CoA, ornithine, putrescine, spermidine, methionine and S-adenosyl L-methionine, some metabolites involved in chitin synthesis, intermediate metabolites of purine metabolism etc (Nurkanto et al., 2018a). Also, it is interesting to note that the transcript levels of DPCK1 increase during encystation stages in *E. invadens* while that of DPCK2 continue to be expressed at comparable levels (Nurkanto et al., 2018a). These findings indicate that these two isoforms have non-overlapping roles in the developmental stages of *Entamoeba*. The work, so far has demonstrated the potential of EhDPCKs as rational drug targets but further screening of chemical libraries and compounds will reveal its true potential.

### Polyamine Biosynthesis

Polyamines are polycations which are aliphatic in nature and have a low molecular weight. They are required by protozoan parasites for their replication (Phillips, 2018). Ornithine decarboxylase (ODC) is the enzyme which catalyses the decarboxylation of ornithine, the rate limiting step in polyamine biosynthesis. Inhibitors of ODC have been used against *Trypanosoma brucei gambiense* (Bottieau and Clerinx, 2019) and *Leishmania donovani* (Kaur et al., 1986) but have been found to be ineffective against ODC of *E. histolytica* as its active site constitutes different amino acid residues (Preeti et al., 2013). So, the focus has been shifted to L-arginase as another target. L-Arginase catalyses the conversion of L-arginine to L-ornithine and this reaction precedes the reaction catalysed by ODC by one step. It also helps the parasite to combat nitrosative stress (Elnekave et al., 2003; Vincendeau et al., 2003; Mortimer and Chadee, 2010) as well as oxidative stress (Shahi et al., 2016) and thus has a significant role in virulence. An inhibitor of L-arginase in *E. histolytica* has been studied which suppresses the growth of trophozoites (Elnekave et al., 2003). Thus, ODC and L-arginase are lucrative drug targets in *E. histolytica* that need further exploration.

## Cellular Drug Targets

### Redox Metabolism

The imbalance between reactive oxygen species (ROS) and the antioxidant defence system in *E. histolytica* causes oxidative stress which ultimately causes harm to the parasite (Burton and Jauniaux, 2011). Nitric oxide (NO) produced by activated macrophages during host immune defence can oxidize and nitrosylate amebic proteins, inhibit glycolysis and reduce virulence while cytotoxin released from immune cells can kill the trophozoites (Pineda and Perdomo, 2017). Absence of catalase, glutathione reductase, and glutathione peroxidise, all of which are present in other aerotolerant protozoans shows that *E. histolytica* is very unique and has developed some alternative defence strategies (Mehlotra, 1996). The major thiol present in the parasite is L-cysteine which is of low molecular weight as compared to glutathione and is required for growth, virulence and antioxidant defence (Dam et al., 2019). L-cysteine, being the most important antioxidant in *E. histolytica*, can be synthesised or acquired by the parasite from the external medium. The two enzymes involved in the synthesis of L-cysteine in the parasite that have been investigated so far, are serine acetyltransferase (SAT) and cysteine synthase (CS). In a medium lacking cysteine, CS has been found to be essential for growth and survival (Jeelani et al., 2017). The screening of inhibitors for CS1, 2 and 3 indicates their potential as drug targets. Although diacetyl kinamycin C and nanaomycin A have been found to be good inhibitors of CS, their use is not feasible for the treatment of amebiasis owing to their toxicity (Mori et al., 2015). However, pencolide is a promising drug candidate with very low toxicity and better anitamoebic activity (Mori et al., 2018). Hence, inhibiting the synthesis pathway of the endogenous reducing agent L-cysteine would hamper the survival of the parasite in the host gut. To add further, this pathway is absent in mammals, hence it could be exploited for developing drugs against amebiasis.

#### Thiredoxin Metabolism

As mentioned earlier, *E. histolytica* lacks the glutathione reductase enzyme (Fahey et al., 1984; Mehlotra, 1996), it largely relies on the thioredoxin system to combat the damage caused by oxidative stress (Jeelani and Nozaki, 2016). The presence of thioredoxin system in the parasite was first proposed by Arias et al. (2007). The genome of *E. histolytica* contains one thioredoxin reductase (TrxR) gene (Arias et al., 2007; Arias et al., 2012) while there are about 22 thioredoxin (Trx) genes (Duchêne, 2015), out of which only two have been studied (Arias et al., 2007; Arias et al., 2008). Hence, a complex biochemical network functions in the parasite. *E. histolytica* TrxR can utilize both NAD(P)H and NADH as reducing equivalents but its affinity for NAD(P)H is 10 times higher than that for NADH (Alkhalfioui et al., 2007; Cheng et al., 2007; Arias et al., 2008). However, the mechanism of thioredoxin reduction in *Homo sapiens* varies from that in this parasite (Becker et al., 2000; Andrade and Reed, 2015), which forms the basis for developing inhibitors of TrxR. The widely used anti-amebic drug, Mtz and its metabolites have been found to form adducts with Trx and TrxR (Leitsch et al., 2007). This covalent interaction of reduced Mtz with Trx and TrxR causes the inhibiton of the disulphide reducing activity of the Trx/TrxR system while the nitrosoreductase activity remains unaffected (Leitsch et al., 2007). This suggests that the Trx/TrxR system is a good target for developing anti-parasitic agents. The drug auranofin which was discovered during automated high throughput screening of anti-amebic drugs, also inhibits TrxR and blocks the reduction of Trx (Debnath et al., 2012). Thus auranofin causes increased sensitivity of the parasite towards reactive oxygen species mediated killing (Debnath et al., 2012). This drug has also been effective against drug resistant *Giardia lamblia, Plasmodium, Schistosoma mansoni* and *Leishmania donovani* (Andrade and Reed, 2015). Hence, the drug can also be developed as a broad spectrum anti-parasitic agent.

### Novel NADH Kinase

When the microaerophilic parasite *Entamoeba histolytica* begins invading the mucosal layer and epithelial tissue, it is attacked by reactive oxygen species. These ROS can damage DNA, proteins and lipids and cause oxidative stress in the parasite (Rastew et al., 2012). NAD(H) kinase is a key enzyme in *E. histolytica* which maintains the levels of NAD(H)/NADP(H) (Pollak et al., 2007). For this, it uses ATP and inorganic phosphate as substrates and plays an important role in tackling oxidative stress. It shows only 20% sequence identity to the NAD kinase of *M. tuberculosis* (Garavaglia et al., 2004), 15% to that of *E. coli* (Kawai et al., 2001) and 18% to that of humans (Lerner et al., 2001). However, it shows highest amino acid sequence identity to NAD(H) kinase from *Trypanosoma brucei* and *Leishmania* major which is 88% for the former and 85% for the latter (Jeelani et al., 2013). A study involving overexpression of NAD(H) kinase showed increased production of NADP^+^ and NADPH and up to 40% decrease in intracellular levels of ROS (Jeelani et al., 2013). Moreover, the trophozoites silenced for the expression of this kinase were not able to survive which indicates its essential status in amebic biology (Jeelani et al., 2013). As a key enzyme in the preservation of NADP^+^ concentration and the maintenance of oxidative stress, it qualifies the status of a potential drug target for amebiasis.

### Chitinase

One of the features of *E. histolytica* is the formation of infectious cysts which have a thick chitin wall. It is made up of chitin fibrils and lectins, and protects the cysts from the harsh conditions of the external environment as well as from the acidic secretions of the host stomach (Samuelson et al., 2013). When the parasite faces nutrient deficiency, it produces cysts and thus manages to come out with the stool in the external environment in order to infect a new host (Ali et al., 2012). Studies on the encystation of *E. histolytica* are still quite an ordeal as it is hard to make the trophozoites encyst in axenic cultures (Mi-ichi et al., 2016). The reptilian parasite *Entamoeba invadens* serves as a model organism for encystation studies as it is closely related to *E. histolytica* and causes the same disease in reptiles (Eichinger, 1997). *E. invadens* carries four active enzymes EiCHT1, EiCHT2, EiCHT3 (Villagómez-Castro and López-Romero, 1996) and EiCHT4 (Makioka et al., 2011) while *E. histolytica* has only one operative enzyme EhCHT1 which is tremendously similar to the homologs in *E. invadens*. The recombinant EhCHT1 was found to be stable over a broad range of temperature and pH while being sensitive to mild concentrations of guanidine hydrochloride (Muñoz et al., 2016). Allosamidin, a substrate analogue inhibitor of chitinase has been found to impede encystation in *E. invadens* and thus, it can be assumed that the inhibition of EhCHT1 would cease the life cycle of the parasite (de la Vega et al., 1997). Targeting key enzymes of encystation and excystation would inactivate pre-mature cysts, thereby preventing the spread of infection.

### Cysteine Proteases (CPs)

About 50 cysteine protease coding genes are present in the *E. histolytica* genome (Irmer et al., 2009). CPs have various functions in amoebic biology like acquiring nutrients (Que and Reed, 2000), degrading mucosal layer (Lidell et al., 2006) and extracellular matrix (Schulte and Scholze, 1989; Horstmann et al., 1992), destroying secretory immunoglobulins (Que and Reed, 2000) and providing resistance against complement mediated lysis (Begum et al., 2015). CP1, 2, 5, and 7 are highly expressed constitutively in trophozoites while CP4 expression is induced upon interaction with mucin producing goblet cells (He et al., 2010). CPs have also been found to play a role in encystation and excystation (Ebert et al., 2008). The amebic CPs are effectively inhibited by E-64 (Sajid and McKerrow, 2002; Olivos-García et al., 2004), a compound from the epoxysuccinate family, which is cell permeable (Satoyoshi, 1992). The CP1 inhibitors K11777 and WRR483, and the CP4 inhibitor WRR605 have been reported to reduce pathogenesis by the parasite in SCID mouse-human colon xenograft model (Meléndez-López et al., 2007). However, the safety and effectiveness of these compounds is yet to be assessed (Nagaraja and Ankri, 2019). Designing specific CP inhibitors is a challenging task as these enzymes are very similar (Que and Reed, 2000). Inhibition of CPs is a very efficient way to prevent the invasion by the parasite in the host tissues (Sajid and McKerrow, 2002). Targeting particularly those CPs whose secretion is induced upon interaction with the host tissue seems a logical way to target parasite invasion. The similar structure of these enzymes would allow a single compound to block a specific set of CPs which is essential for pathogenesis.

### Protein Kinases

The kinome of *E. histolytica* constitutes about 3.7% of its total proteome which is more than that of most other eukaryotes (Ahmad et al., 2020). The genome of *E. histolytica* encodes 307 putative kinases (Anamika et al., 2008) which is less than half the number of kinases in the human kinome (Manning et al., 2002) and thrice the number of kinases found in the malaria parasite, *Plasmodium falciparum* (Ward et al., 2004). Such a large kinase repertoire is unexpected for a single celled eukaryote. As already mentioned, endocytic processes are essential for survival and virulence of the parasite (Meza and Clarke, 2004). The parasite exhibits phagocytosis which is meant for engulfing dead host cells, RBCs and bacteria (Iyer et al., 2019), while live host cells are ingested through a nibbling process known as trogocytosis (Ralston, 2015b). Besides this, trophozoites take up fluid through pinocytosis and micropinocytosis (Laughlin et al., 2004). Many molecules involved in endocytic processes of the parasite are known and have been characterized. Molecules like Gal/GalNAc lectin, EhCaBP1, 3, 5, actin, Arp2/3 (Christy and Petri, 2011), Rho1 (Bosch et al., 2012), Rabs (Verma et al., 2020) and coactosin (Hasan et al., 2020) are few to be mentioned which have been characterized. Apart from these, a number of kinases like EhC2PK (a C2 domain containing kinase) (Somlata et al., 2011), EhAK1 (an atypical alpha kinase) (Mansuri et al., 2014) and EhAGCK1 (an AGCK family kinase) (Somlata et al., 2017) also play an important role in endocytic processes at various stages like initiation of phagocytosis, polymerisation of actin and phagocytosis progression. These kinases have been shown to be crucial for amebic biology and their downregulation inhibits the proliferation and thus the survival of the parasite. EhC2PK is involved in initiation of phagocytic cups and is recruited to the site of phagocytosis in a Ca^2+^-dependent manner. The downregulation of kinase expression in trophozoites *via* anti-sense RNA expression under an inducible system leads to growth defects and a decrease in phagocytosis (Somlata et al., 2011). EhC2PK is also involved in trogocytosis which involves nibbling of live host cells by the parasite (Ralston et al., 2014). Hence, it appears that this kinase is one of the fundamental proteins involved in the initiation of actin dependent endocytic processes. The kinase domain of EhC2PK is similar to CaM kinases along with a C2 domain at its N-terminal, which is an unusual composition (Somlata et al., 2011). This type of kinase involvement in endocytic processes is unique to *E. histolytica* and has not been reported elsewhere. Following the initiation of the phagocytic cup, actin remodelling and polymerisation occur so as to provide the necessary force for pulling the membrane inward in order to internalise the target cell/particle. On the other hand, EhAK1 is a kinase which is involved in phosphorylation of actin and Arp2/3 subunit (Mansuri et al., 2014). Arp2/3 is also recruited by this kinase to site of phagocytosis which is important for initiating actin polymerisation. The phosphorylation of actin by this enzyme further promotes actin polymerisation (Babuta et al., 2015). The downregulation of kinase expression and overexpression of non-functional kinases lead to defects in phagocytosis (Mansuri et al., 2014). Various experimental studies indicate EhAK1 to be downstream of EhC2PK, coupling Ca^2+^ signalling and actin dynamics at the site of phagocytosis (Babuta et al., 2020). Apart from these, a conserved AGCK family kinase, EhAGCK1, has been shown to be exclusively involved in trogocytosis. This kinase has an N-terminal PH domain followed by a kinase domain which is similar to AktB in humans. The kinase is recruited to the cytosolic side of the membranes of narrow tunnel-like structures formed during nibbling of live host cells in a PIP_3_-dependent manner (Somlata et al., 2017). Since the kinase is exclusively involved in trogocytosis, it can be targeted for blocking host tissue invasion by the parasite. EhC2PK, EhAK1 and EhAGCK1 have been studied for their role in amebic biology but none of them have been screened for inhibitors against any chemical libraries. However, alpha kinase has shown to be inhibited in the soil dwelling amoeba *Dictyostelium discoideum* resulting in many defects such as impaired development, multinucleation and growth retardation (Betapudi et al., 2005). Thus the crucial roles and diverged sequences of these biomolecules make them eligible to be considered as promising drug targets.

### Transcription Factors

Transcription factors (TF) are the key regulators of gene expression and are capable of binding to complex DNA sequences and controlling gene expression (Spitz and Furlong, 2012). The TF Encystation Regulatory Motif - Binding Protein (ERM-BP) was found to play a key role in regulating encystation in *E. histolytica* and its downregulation led to low encystation activity and the formation of faulty cysts (Manna et al., 2018). ERM-BP directly binds to the coenzyme NAD^+^ which increases during encystation and modifies the factor’s conformation for promoter binding (Manna et al., 2018). Overexpressed ERM-BP defends both, *E. invadens* and *E. histolytica* against death from heat shock and promotes the formation of multinucleated giant cells (MGC) which develop from trophozoites during encystation, while silencing of ERM-BP leads to a reduction in the formation of MGC (Manna et al., 2020). Since ERM-BP is involved in encystation, inhibiting it would prevent cyst formation, thereby decreasing the chances of infection in another host as cysts are the infective form of the parasite.

### Calcium Signalling

*Entamoeba histolytica* shows the presence of extensive calcium signalling system which is thought to be involved in invasion and pathogenesis by the parasite. The genome of *E. histolytica* encodes 27 EF-hand containing calcium-binding proteins (CaBPs) (Bhattacharya et al., 2006) and 36 putative CaM kinases. However, these putative CaM kinases are not like typical CaM kinases and show only 30% sequence identity in their catalytic region to those in *Homo sapiens* and hence, can be called CaM-like kinases (Anamika et al., 2008). In AmoebaDB, some of the CaM kinases are reported in the phagosome proteome of *E. histolytica* which indicates their possible role in endocytic processes. It has been shown that calcium chelation by BAPTA-AM, an intracellular Ca^2+^ chelator, reduced phagocytosis by 60% which proves that calcium plays an important role in pathogenesis by *E. histolytica* (Jain et al., 2008). Some of the CaBPs of *E. histolytica* - EhCaBP1, 3 and 5 are involved in endocytic processes *via* actin remodelling and recruitment of other downstream proteins. EhCaBP3 (Aslam et al., 2012) and EhCaBP5 (Kumar et al., 2014), both have been shown to interact with MyosinIB and their Ca^2+^ binding property is essential for their functioning *in vivo*. The C2 domain of EhC2PK, which plays a key role in phagocytosis (Somlata et al., 2011) as well as in trogocytosis (Ralston et al., 2014), binds to the membrane in the presence of Ca^2+^ while EhAK1 interacts with EhCaBP1 only when Ca^2+^ ions are present (Babuta et al., 2020). All these proteins play a primary role in the initiation of phagocytosis and thus hijacking the Ca^2+^ signalling network might prove to be of great therapeutic value for drug development. However, this avenue needs more detailed investigation.

### COP9 Signalosome

COP9 signalosome is the cellular machinery responsible for protein degradation and is present in numerous protozoan parasites including *E. histolytica*, *Toxoplasma*, *Leishmania* and *Trypanosoma* (Field et al., 2017; De Rycker et al., 2018; Shirley et al., 2019). It acts as an upstream regulator of the ubiquitin-proteasomal system (UPS) which is present in all eukaryotes and is also involved in protein degradation (Xie et al., 2019). Recently, in *E. histolytica*, the subunit 5 of COP 9 signalosome, CSN5 (also known as COPS5 and JAB1) has been characterized and identified as the catalytic centre of the COP9 signalosome. CSN5 (EHI_050500) contains a JAMM (JAB1/MPN/Mov34 metalloenzyme) motif. Other components of the signalosome complex such as CSN2 (EHI_174890), CSN1 (EHI_182890), CSN3 (EHI_103560), CSN6 (EHI_068470) were also co-purified while putative genes for CSN4 and CSN8 were identified in the *E. histolytica* genome (Ghosh et al., 2020). In the same study, the CSN5 gene was silenced using high-efficiency inducible RNA interference technology to develop knockout trophozoites which exhibited reduced proliferation and were eventually killed. Expression of dominant negative mutant of CSN5 also showed the same results (Ghosh et al., 2020). Also, the drug zinc-ditiocarb (ZnDTC) was tested *in vitro* against wild type trophozoites and it was found to reduce their viability. The drug was even tested *in vivo* using mouse models that mimicked human amebic colitis and showed positive results for pathogen clearance (Ghosh et al., 2020). The ZnDTC is already an FDA approved drug which is used for treating alcoholism and can be repurposed as an anti-amebic agent. Thus, CSN5 and other components of the COP9 signalosome complex in *E. histolytica* are excellent potential drug targets.

### Peptidyl Transferase Activity of the 60S Ribosomal Subunit

Small cohort studies for the drug anisomycin to treat amebiasis have previously been done (Gonzalez Constandse, 1956). The drug acts by inhibiting the peptidyl transferase activity of 60S ribosomal subunit (Macias-Silva et al., 2010). Other inhibitors of this enzyme - puromycin, amicetin and blasticidin S have been studied in *Dictyostelium discoideum* which affected its tRNA maturation (Stathopoulos et al., 2000). To add further, the translation machinery of *Plasmodium falciparum* is also inhibited by these inhibitors and has been considered as an attractive drug target in the parasite (Sheridan et al., 2018). Hence, peptidyl transferase is also a potential drug target in *E. histolytica*, whose inhibition would suppress all protein synthesis processes of the parasite.

### β-Amylase

*E. histolytica* secretes β-amylase which helps it in degrading MUC2 mucin in the mucosal layer of the large intestine during invasion (Thibeaux et al., 2013). Since humans lack β-amylase, it is an excellent drug target as its inhibition can be useful in controlling invasive form of the amebiasis.

### Candidates for Developing Anti-Amebic Vaccine

#### Gal/GalNAc Lectin

So far, no effective vaccine has been developed against amebiasis however, researchers in the course of time have identified biomolecules for vaccine development. One of the candidates is Gal/GalNAc (galactose/N-acetyl galactosamine) lectin which is present on the extracellular side of the plasma membrane of *E. histolytica*. There is a 260 kDa lectin and a 150 kDa lectin in the parasite which associate with each other in order to carry out their function (Mann, 2002). Upon coming in contact with galactose or N-acetyl galactosamine residues on the surface of host cells such as RBCs, the lectins participate in a signalling cascade which leads to the endocytosis of the host cell by the parasite (Christy and Petri, 2011). Gal/GalNAc lectins are also involved in adherence, invasion, cytolysis and evasion from complement-mediated lysis due to which they are crucial for virulence (Mann, 2002). Vaccines based on Gal/GalNAc lectin have shown to confer mild to full protection in many animals such as gerbils and mice (Singh et al., 2016). The most effective results have been obtained with four polylysine-linked synthetic peptide vaccine prepared using the heavy chain of Gal/GalNAc lectin in which the adjuvant was cholera toxin (Abd Alla et al., 2012). Therefore, Gal/GalNAc lectin is another potential drug target which upon inhibition would make the parasite unable to carry out endocytosis, putting its survival at stake.

#### SREHP

Another protein which has been investigated for generating vaccine is the serine rich *Entamoeba histolytica* protein (SREHP) which is a plasma membrane protein. It aids Gal/GalNAc lectin in adhesion of trophozoites to host cells, cell killing and eventual phagocytosis (Teixeira and Huston, 2008). Its inhibition has shown to reduce lectin-independent phagocytosis of apoptotic Jurkat lymphocytes along with a reduction in adherence and killing of viable Jurkat lymphocytes. This was done by using monoclonal antibodies developed in mice against the surface antigens of trophozoites. Out of all the screened antibodies, one antibody, 10D11Ab was able to bind to SREHP and block its activity (Teixeira and Huston, 2008). Also, DNA vaccines containing plasmids encoding for SREHP have shown promising results in gerbils and mice (Zhang and Stanley, 1999). About 60% of vaccinated gerbils were protected against amebic liver abscesses while protection in vaccinated mice against liver abscess was 80% (Zhang and Stanley, 1999). Thus, SREHP is another promising drug target whose inhibition would render the trophozoites unable to cause adhesion, cell killing and phagocytosis. This strategy of protection against the parasite is highly useful for international travellers visiting endemic regions. Although people residing in endemic regions can also be benefitted from vaccination, long term protection has not been assessed with available options.

### Harnessing the Potential of Gut Microbes

*E. histolytica* trophozoites survive on bacterial cells and cellular debris in the host gut until they turn virulent (Galván-Moroyoqui et al., 2008; Travers et al., 2011; Varet et al., 2018). It has also been observed that selective bacterial strains are ingested by trophozoites (Schulz et al., 1987). Also, the interaction with specific intestinal bacteria influences the parasite’s cell surface characteristics which further changes the paradigm of interactions carried out by the trophozoites in the gut environment  (Bhattacharya et al., 1992; Ankri et al., 1999). Moreover, the gut microbiome of patients suffering from amebiasis in comparison to normal ones differs in terms of decreased populations of *Bacteroides*, *Clostridium*, *Lactobacillus*, *Campylobacter*, and increased population of *Bifidobacterium*  (Verma et al., 2012). Hence, deviation from normal gut microbiota might trigger or aid the pathogenesis by this parasite  (Nagaraja and Ankri, 2019). Also, there has been a positive co-relation between oxaloacetate (OAA) producing bacteria with high *E. histolytica* burden as OAA protects trophozoites against oxidative stress and promotes virulence  (Shaulov et al., 2018). Another bacteria, *Prevotella copri*, is also associated with gut inflammation and high load of *E. histolytica*  (Gilchrist et al., 2016; Larsen, 2017). The interaction of *E. histolytica* with gut microbiome can be harnessed to render the parasite avirulent and protect the host  (Nagaraja and Ankri, 2019). One of the most well-known approach is the use of probiotics. These are concoctions of live bacteria which restore the healthy gut microbiome  (Galván-Moroyoqui et al., 2008; Goyal et al., 2011; Hill et al., 2014; Sarjapuram et al., 2017). Probiotics act through a range of mechanisms like competing with the pathogen for binding sites on host mucosal surface, reducing adhesion of the parasite on intestinal mucosal surface, producing anti-microbial products and competing for nutritional substrate in the gut lumen  (Travers et al., 2011). Alteration in food habits coupled with the use of probiotics  (Kumar et al., 2015) can help in restoring gut microbiome which is protective against parasitic infections and pathogenesis.

## Conclusion

**Figure 2:**
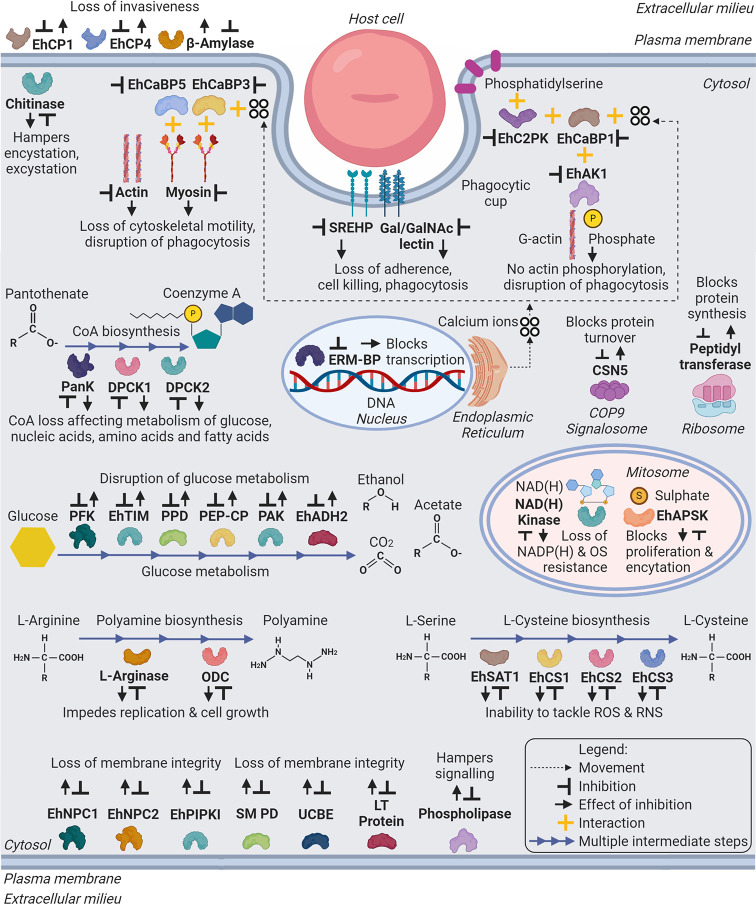
Schematic representation of major drug targets (labelled in bold) in *Entamoeba histolytica*. The representation includes metabolic and cellular pathways, which can be target of developing therapeutic agents against amebiasis. Abbreviations: Eh - *E. histolytica*; EhCP1,4 - Cysteine Protease 1, 4; EhCaBP5,3,1 - Calcium Binding Protein 5,3,1; EhC2PK - C2 domain containing Protein Kinase; EhAK1 - Alpha Kinase 1; SREHP - Serine Rich *E. histolytica* Protein; Gal/GalNAc lectin – Galactose/N-Acetylgalactosamine lectin; PanK or EhPanK - Pantothenate Kinase; DPCK1,2 or EhDPCK1,2 - Dephospho-CoA Kinase 1,2; ERM-BP - Encystation Regulatory Motif-Binding Protein; CSN5 - COP9 Signalosome Subunit 5; PFK - Phosphofructokinase; EhTIM - Triosephosphate Isomerase; PPD - Pyruvate Phosphate Dikinase; PEP-CP - Phosphoenolpyruvate Carboxytransphosphorylase; PAK - Pyrophosphate Acetate Kinase; EhADH2 - Alcohol Dehydrogenase 2; NAD(H) kinase - Nicotinamide Adenine Dinucleotide (Hydride) kinase; ODC or EhODC - Ornithine Decarboxylase; EhSAT1 - Serine Acetyltransferase 1; EhCS1,2,3 - Cysteine Synthase 1,2,3; EhNPC1,2 - Niemann-Pick type C protein 1,2; EhPIPKI - PI(4)P [Phosphatidylinositol 4-phosphate] Kinase I; Putative SM PD - Putative Sphingomyelin Phosphodiesterase; UCBE - Uncharacterized Ceramide Biosynthesis Enzyme(s); LT Protein - Lipid Transfer Protein(s). (Created with BioRender.com).

In recent times, emphasis on and widespread publicity of clean drinking water, personal hygiene and diagnosis has drastically reduced the oral-faecal transmission of amebiasis along with other diseases, especially, in the developed countries. But in developing countries, particularly rural regions, the incidence of infection is still high due to lack of potable water and poor hygiene awareness. The treatment of amebiasis majorly depends on Mtz which is a cost effective and easily available drug. However, its potential genotoxic and neurotoxic side effects along with the likely emergence of Mtz-resistance are the reasons to search for alternate lines of treatment for amebiasis. The new lines of treatment might emerge, firstly by repurposing already existing drugs like auranofin which is used for treating arthritis. Such drugs have already been approved with data regarding their toxicity, safety and pharmacokinetics in humans which would shorten the time for clinical application after their anti-amebic or anti-parasitic activity has been assessed. Secondly, the pathways which are crucial and unique to its system such as thiol-based redox metabolism, processes involved in encystations etc can be identified and targeted for drug development. Although, *E. histolytica* resides in a rapidly changing intestinal environment and has mechanisms to adapt efficiently, its cellular and metabolic processes differ from those of its host. This can be exploited to identify drug targets and develop new drug molecules against the parasite. As already mentioned, *E. histolytica* is microaerophilic and uses L-cysteine as the major antioxidant against oxidative stress. More detailed investigations and research might lead to development of alternate anti-amebic drugs. Another unique metabolic pathway of sulphur metabolism is a very promising target as its inhibition leads to defective encystation. Inhibition of these targets would provide dual benefits - ceasing the proliferation of trophozoites in the host as well as the dissemination of cysts in the environment, thereby lowering the infection rate. Another promising approach which can be pursued is, targeting the pathways related to invasive amebiasis. Motility and endocytosis, particularly phagocytosis and trogocytosis are important for virulence of the parasite. Destruction of host tissues is mediated primarily through phagocytosis and trogocytosis which seem to have overlapping molecular mechanisms. However, the kinases known so far, like EhC2PK, EhAK1 and EhAGCK1 which are involved in processes unique to *E. histolytica* offer to be attractive targets for drug development as they are very essential for the endocytosis of dead or live host cells. So far, the potential of these kinases has not been explored but considering them will be worthwhile in the the course of developing anti-amebic drugs. The major drug targets have been summarised in **Table 1** and schematically represented in **Figure 2**. Besides this, a lot of details about mechanisms of pathogenic processes are yet to be discovered which means that in due course of drug development, new drug targets would be discovered which might be related to actin dynamics, membrane remodelling and/or small GTPases. Nevertheless, it is very encouraging to see that a numerous libraries of chemical and natural compounds have been screened against *E. histolytica* and many potential natural and synthetic anti-parasitic molecules have been identified. However, they need to be characterized in more detail in terms of their toxicity, mechanism of action, safety and pharmacokinetics before clinical applications. Along with the development of alternative treatments for amebiasis, it will be worthwhile to work on preventive strategies like vaccination and the use of probiotics. Although, the work in this field, so far has not reached to clinical applications but it would hugely benefit international travellers and people residing in endemic countries.

**Table 1:** Drug Targets in *Entamoeba histolytica*.

S. No.	Drug target	Role	Inhibited in *E. histolytica via* any drug or molecule?	Inhibited in *E. histolytica via* gene silencing or downregulation?	Inhibited in any other organism?	Other organism(s) or cell line(s) in which inhibition was studied*	References
1.	Nicotinamide Adenine Dinucleotide (Hydride) Kinase (NAD(H) kinase)	Production of NADP(H) and NAD(H) and tackling oxidative stress	No	Yes	Yes	*Mycobacterium tuberculosis*	(Kawai et al., 2001; Lerner et al., 2001; Garavaglia et al., 2004; Jeelani et al., 2013)
2.	Encystation Regulatory Motif - Binding Protein (ERM-BP)	Regulation of encystation	No	Yes	No	–	(Manna et al., 2018; Manna et al., 2020)
3.	C2 domain containing Protein Kinase (EhC2PK)	Initiation of phagocytosis	No	Yes	No	–	(Somlata et al., 2011)
4.	Alpha Kinase 1 (EhAK1)	Actin phosphorylation during phagocytosis	No	Yes		*Dictyostelium discoideum*	(Mansuri et al., 2014; Babuta et al., 2020)
5.	Chitinase	Encystation	No	Yes	Yes	*Entamoeba invadens*	(de la Vega et al., 1997; Muñoz et al., 2016)
6.	Alcohol Dehydrogenase 2 (EhADH2)	Glucose metabolism	Yes	Yes	Yes	*Escherichia coli*	(Yang et al., 1994; Yong et al., 1996; Espinosa et al., 2001; Pineda et al., 2015; Espinosa et al., 2016)
7.	Adenosine 5’-phosphosulphate Kinase (EhAPSK)	Sulphur metabolism	Yes	Yes	Yes	T*oxoplasma gondii, Plasmodium falciparum, Giardia intestinalis, Trypanosoma brucei, Trypanosoma cruzi*	(Mi-ichi et al., 2009; Makiuchi and Nozaki, 2014; Mi-ichi et al., 2015; Mi-ichi et al., 2017; Mi-ichi et al., 2019)
8.	Ornithine Decarboxylase (ODC)	Decarboxylation of ornithine (rate limiting step in polyamine biosynthesis)	No	No	Yes	*Trypanosoma brucei gambiense, Leishmania donovani*	(Kaur et al., 1986; Bottieau and Clerinx, 2019)
9.	Serine Acetyl Transferase (EhSAT1)	Combatting oxidative stress, L-cysteine biosynthesis pathway: conversion of L-serine into acetyl-L-serine	No	No	Yes	*Escherichia coli*	(Agarwal et al., 2008; Jeelani et al., 2017)
10.	22 Lipid transfer proteins	Processing of exogenous lipids	No	No	Yes	HeLa cells	(Das et al., 2002; Das and Nozaki, 2018; Nakao et al., 2019)
11.	L-Arginase	Polyamine biosynthesis - conversion of L-arginine to L-ornithine	Yes	No	Yes	*Leishmania major*, *Leishmania infantum*	(Iniesta et al., 2001; Elnekave et al., 2003)
12.	Cysteine Synthases (CS1, CS2, CS3)	L-Cysteine biosynthesis, conversion of acetyl-L-serine to L-cysteine, combating oxidative stress	Yes	No	Yes	*Mycobacterium tuberculosis*	(Mori et al., 2015; Brunner et al., 2016)
13.	Cysteine Proteases (EhCP1 and EhCP4)	Proteolysis, degradation of extracellular matrix and mucosal layer, encystations and excystation, erythrophagocytosis etc	Yes	No	Yes	*Trypanosoma brucei, Trypanosoma cruzi, Plasmodium* sp.	(Meléndez-López et al., 2007; He et al., 2010; Siqueira-Neto et al., 2018)
14.	Phosphofructokinase	Glucose metabolism: phosphorylation of fructose-6-phosphate	Yes	No	Yes	*Toxoplasma gondii*	(Eubank and Reeves, 1982; Peng et al., 1995; Bruchhaus et al., 1996)
15.	Phosphoenolpyruvate Carboxytransphosphorylase	Glucose metabolism	Yes	No	Yes	*Propionibacterium*	(Utter and Kolenbrander, 1972; Mertens, 1993; Varela-Gómez et al., 2004; Chiba et al., 2015)
16.	Pyruvate Phosphate Dikinase	Glucose metabolism	Yes	No	Yes	*Flaveria trinervia*	(Mertens, 1993; Varela-Gómez et al., 2004; Chiba et al., 2015; Minges and Groth, 2017)
17.	Pyrophosphate-acetate Kinase	Glucose metabolism	Yes	No	No	–	(Mertens, 1993; Varela-Gómez et al., 2004; Chiba et al., 2015)
18.	Pantothenate Kinase	CoA synthesis required for fatty acid synthesis	Yes	No	Yes	*Mycobacterium tuberculosis, Bacillus anthracis, Klebsiella pneumonia*, *Staphylococcus aureus*	(Reddy et al., 2014; Hughes et al., 2014; Nurkanto et al., 2018a; Nurkanto et al., 2018b; Shapiro et al., 2019)
19.	Peptidyl Transferase	Protein Synthesis	Yes	No	Yes	*Dictyostelium discoideum*, *Plasmodium falciparum*,	(Stathopoulos et al., 2000; Gonzalez Constandse, 1956; Sheridan et al., 2018)
20.	Sphingomyelin Phosphodiesterase and one similar enzyme	SL metabolism and Kennedy pathway	No	No	Yes	L929 cell line, HepG2 cell line and B16F10 cell line, human lung cells, mouse lung cells, *Plasmodium falciparum*	(Hanada et al., 2000; Justice et al., 2018; Castellanos-Castro et al., 2020; Naser et al., 2020)
21.	Uncharacterized enzymes in ceramide biosynthesis	Ceramide biosynthesis	No	No	Yes	*Dictyostelium discoideum*, *Plasmodium falciparum*, *Plasmodium knowlesi*	(Min et al., 2005; Meyer et al., 2012; Njoya et al., 2014)
22.	PI(4)P [Phosphatidylinositol 4-phosphate] Kinase I (EhPIPKI)	Synthesis of PI(4,5)P2; parasite motility	Yes	No	Yes	*Plasmodium falciparum*	(Byekova et al., 2010; Vaid et al., 2010; Das and Nozaki, 2018; Sharma et al., 2019)
23.	Phospholipase	Synthesis of signaling molecules, parasite motility	Yes	No	Yes	*Dictyostelium discoideum*	(Long-Krug et al., 1985; Bominaar and Van Haastert, 1994; Meza, 2000; Kortholt et al., 2007)
24.	Niemann-Pick type C Proteins (EhNPC1 and 2)	Cholesterol transport	Yes	Yes	Yes	*Plasmodium falciparum*	(Bolaños et al., 2016; Istvan et al., 2019)
25.	EhDPCK1 and 2	Metabolism of CoA, purines, chitin	Yes	No	Yes	*Plasmodium falciparum*	(Fletcher et al., 2016; Nurkanto et al., 2018a)
26.	COP9 Signalosome Subunit 5 (CSN5) and other subunits	Upstream regulator of ubiquitin-proteasomal system	Yes	Yes	Yes	*Trypanosoma cruzi, Leishmania* spp.*, Trypanosoma brucei* spp.	(Khare et al., 2016; Ghosh et al., 2020)
27.	β-Amylase	Degradation of MUC2 mucin in the mucosal layer, helps in invasion	No	Yes	Yes	*E. invadens, Arabidopsis*	(Serafimova, 1996; Umar Dahot et al., 2004; Thibeaux et al., 2013; Nakada-Tsukui and Nozaki, 2016; Li et al., 2017)
28.	Gal/GalNAc Lectin	Adhesion, cell killing, phagocytosis	Yes	No	Yes	*Hartmannella vermiformis*	(Venkataraman et al., 1997; Abd Alla et al., 2012; Aguirre García et al., 2015; Singh et al., 2016)
29.	Serine Rich *Entamoeba histolytica* Protein (SREHP)	Adhesion, cell killing, phagocytosis	Yes	No	No	–	(Zhang and Stanley, 1999; Teixeira and Huston, 2008)

*This column does not include all the organisms other than E. histolytica in which inhibition has been studied.

## Author Contributions

MTS and ZM reviewed the literature and collected all the information. MTS and ZM also drafted the manuscript. S directed the manuscript writing process and drafted the plan of manuscript outline. All authors contributed to the article and approved the submitted version.

## Conflict of Interest

The authors declare that the research was conducted in the absence of any commercial or financial relationships that could be construed as a potential conflict of interest.
